# Infective endocarditis following tumor necrosis factor-α antagonist therapy for management of psoriatic erythroderma: a case report

**DOI:** 10.1186/s13256-016-1130-1

**Published:** 2017-02-09

**Authors:** Takuro Mizuno, Jun Kiyosawa, Akihiro Fukuda, Seiji Watanabe, Nozomu Kurose, Takayuki Nojima, Tsugiyasu Kanda

**Affiliations:** 1 Department of Cardiovascular Medicine, Kanazawa Medical University Himi Municipal Hospital, 1130 Kurakawa, Himi, Toyama 935-8531 Japan; 2 Department of Dermatology, Kanazawa Medical University Himi Municipal Hospital, 1130 Kurakawa, Himi, Toyama 935-8531 Japan; 3 Department of Pathology and Laboratory Medicine, Kanazawa Medical University, 1-1 Uchinada, Ishikawa, 920-0293 Japan; 4 Department of Community Medicine, Kanazawa Medical University Himi Municipal Hospital, 1130 Kurakawa, Himi, Toyama 935-8531 Japan

**Keywords:** TNF-α antagonist, Psoriasis erythroderma, Bacterial endocarditis

## Abstract

**Background:**

The introduction of biological agents, such as infliximab, which act against tumor necrosis factor-α was a major advance for the treatment of an increasing number of chronic diseases. Tumor necrosis factor-α antagonists represent a major therapeutic advance for the management of chronic inflammatory diseases, such as psoriasis. Previous studies have reported that the use of tumor necrosis factor-α antagonists increased the risk of opportunistic infections and reactivation of latent bacterial infections. Cardiac involvement, such as infective endocarditis, is very rare in the literature.

**Case presentation:**

A 77-year-old Asian man with a 10-year history of psoriatic erythroderma was referred due to high fever and general malaise. He was treated with Predonine (prednisolone) and infliximab. After treatment, cardiac echography showed mitral valve vegetation and brain magnetic resonance imaging indicated multiple fresh infarctions. He died from large brain infarction in October 2013. An autopsy showed fresh thrombosis in his left middle cerebral artery, mitral valve vegetations, and septic micro-embolisms in multiple organs.

**Conclusions:**

Lethal bacterial endocarditis was revealed after administration of tumor necrosis factor-α inhibitor, infliximab, for the treatment of psoriatic erythroderma. An autopsy showed vegetation in his mitral valve and brain infarction with fresh purulent embolism in his left middle cerebral artery and septic micro-embolisms.

## Background

The introduction of biological agents, such as infliximab, which act against tumor necrosis factor-α (TNF-α) was a major advance for the treatment of an increasing number of chronic diseases. TNF-α antagonists represent a major therapeutic advance for the management of chronic inflammatory diseases, such as rheumatoid arthritis and psoriasis [1]. Due to their inhibition of proinflammatory cytokines, their use has been associated with an increased risk of severe infection [2]. Previous studies have reported an increased risk of opportunistic infections and reactivation of latent bacterial infections [3]. Although some studies concluded an increased risk of infection in patients receiving infliximab [4], the relative risk for patients receiving TNF-α inhibitors was reported to be up to twice that of control [5], and even higher at the treatment’s onset. Cardiac involvement, such as infective endocarditis, is very rare in the literature [6, 7].

We report the case of a man with infective endocarditis due to the TNF-α inhibitor, infliximab, for the management of psoriatic erythroderma.

## Case presentation

**Fig. 1:**
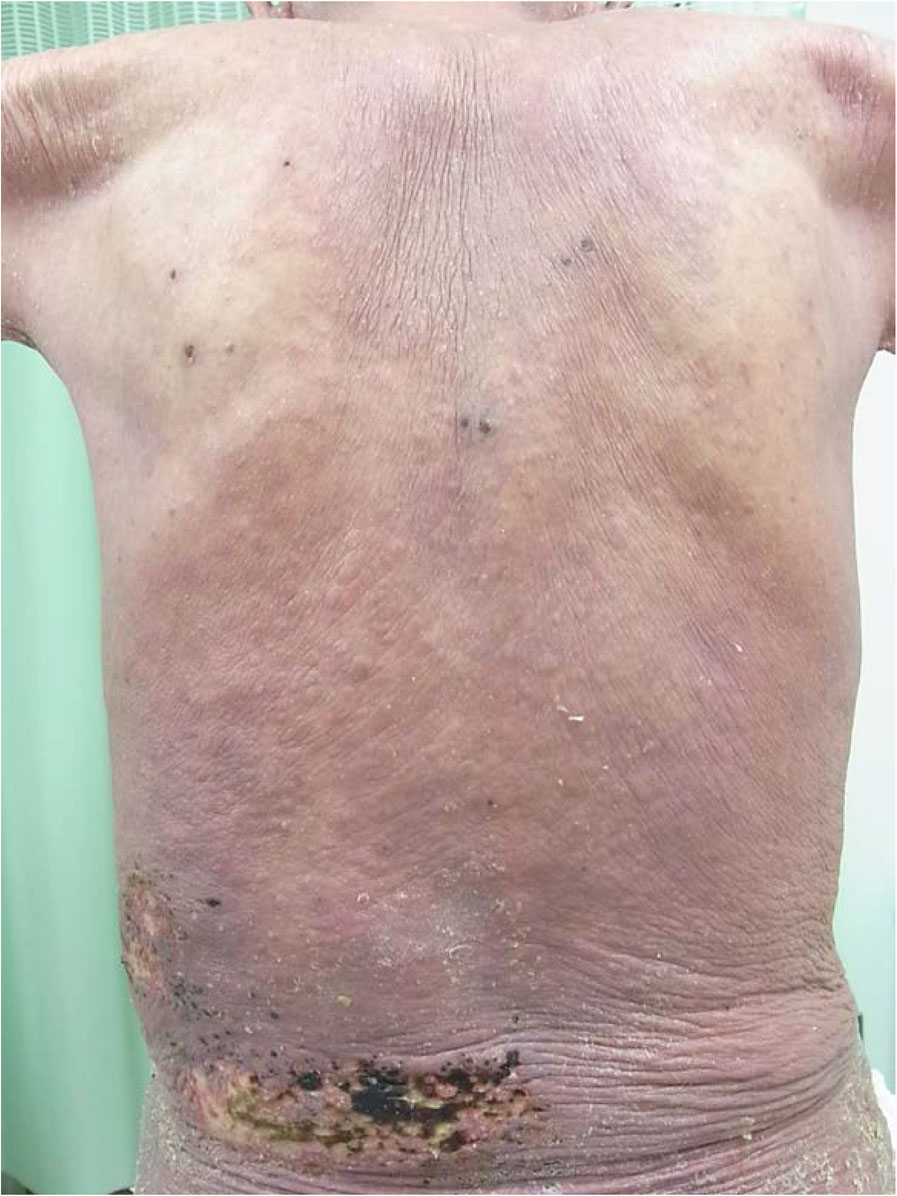
The dorsal surface of trunk showed red-violet erythema and fine scaling with the healed herpes zoster

We describe the case of a 77-year-old Asian man with a 10-year history of moderate-to-severe psoriatic erythroderma (Fig. 1). He was treated with an anti-histamine drug and steroid ointment on his psoriasis for a few years but he was not treated with oral steroid agents. His psoriasis worsened. Considering the clinical severity of the features of his skin, he was treated with 14 days of Predonine (prednisolone) 15 mg orally and one treatment of infliximab 300 mg by intravenously administration in August 2013. He was discharged at the end of August 2013 because his skin condition was better and his itching symptom was relieved.

Three days later, he presented with shortness of breathing, general malaise, and anorexia. He was referred to our department of cardiovascular medicine in September 2013. His past medical history showed that he had herpes zoster 6 months earlier. His social history was not significant for alcoholic drinks or tobacco smoking. He denied illicit drug use. His job was a farmer. No particular environment was evident.

**Fig. 2:**
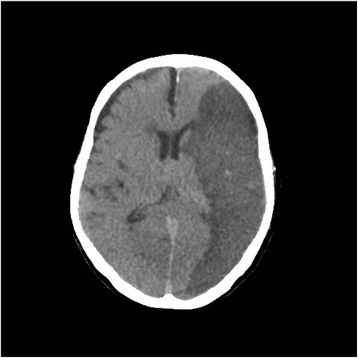
Computed tomography showed the large cerebral infarction on day 29 after admission

**Fig. 3:**
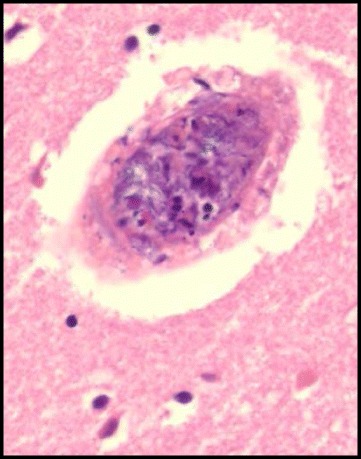
Autopsy revealed the fresh thrombosis in left middle cerebral artery

**Fig. 4:**
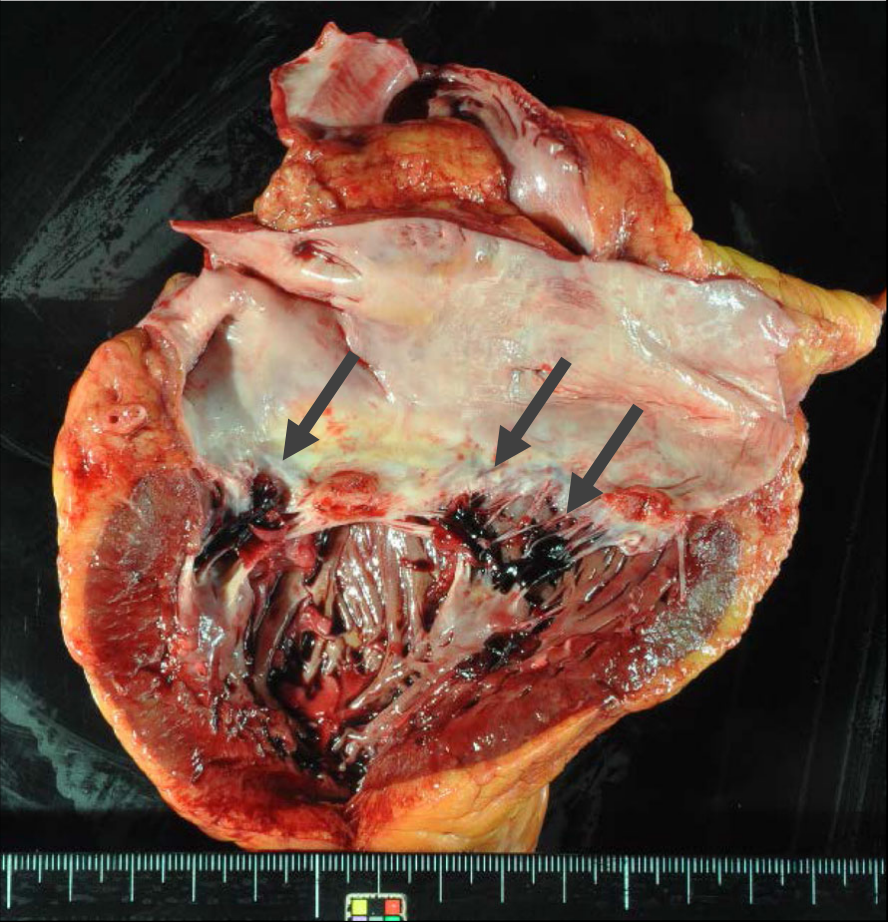
*Arrows* show multiple mitral valve vegetations

On physical examination, he was awake and oriented to time, place, and self. A neurological examination showed no abnormal reflex and no paralysis. His vital signs revealed heart rate of 111/minute, blood pressure of 101/49 mmHg, respiratory rate of 24/minute with an oxygen saturation of 97 % on 3 L/minute nasal oxygen supply. The other physical findings revealed no murmur and no wheezing in his chest and no tenderness in his abdomen. He underwent a series of laboratory tests. His white blood cell (WBC) count was 29,900/mm^3^ and polymorphonuclear neutrophilic cells were 84 %. His levels of liver and renal function were within normal range. Chest X-rays showed slight cardiac enlargement. An electrocardiogram showed preventricular atrial contraction. Chest computed tomography revealed pleural effusion and cardiac enlargement. The first cardiac echography showed mitral valve vegetation with diffuse hypokinesis of left ventricular wall motion and brain magnetic resonance imaging (MRI) indicated multiple fresh infarctions. He was given empiric therapy with piperacillin-tazobactam administered intravenously 4.5 g twice a day. His blood culture revealed methicillin-sensitive *Staphylococcus aureus* infection. His antimicrobial therapy was modified to monotherapy with daptomycin administered intravenously 300 mg/day. Although the regimen was held [8], his systemic conditions worsened and he was unconscious. Disseminated intravascular coagulation and cardiogenic shock progressed. The following cardiac echography showed no mitral valve vegetation with grade III mitral regurgitation. We speculated vegetation removal from his mitral valve. He died 31 days after readmission from large brain infarction (Fig. 2) in October 2013. An autopsy showed fresh purulent embolism in his left middle cerebral artery (Fig. 3), mitral valve vegetations (Fig. 4), and septic micro-embolisms in multiple organs.

## Discussion

We described a patient with lethal bacterial endocarditis after administration of TNF-α inhibitor, infliximab, for the treatment of psoriatic erythroderma. An autopsy showed vegetation in his mitral valve and brain infarction with fresh purulent embolism in his left middle cerebral artery and septic micro-embolisms.

Erythrodermic psoriasis is a severe variant of psoriasis with a reported prevalence among patients with psoriasis ranging from 1.5 to 31 % [9]. Left untreated, it may lead to serious morbidity and even mortality. Erythrodermic psoriasis is often difficult to manage, and the therapies that are currently available may prove to be unsatisfactory. Biological agents offer a new alternative therapeutic approach [9], although there are no controlled trials to support their use, particularly as a long-term therapy option. The relation between erythrodermic psoriasis and cardiac disease is only mentioned in coronary artery disease. In many ways, psoriasis can be considered a model autoimmune disease [10]. The most common causes of death in patients with erythroderma are pneumonia, septicemia, and heart failure. Older patients who develop complications such as infection, fluid/electrolyte imbalances, and cardiac failure are at higher risk of mortality [11]. In our case, septic organ failure due to treatment-induced bacterial endocarditis was the cause of death.

Bacteremia due to receiving TNF-α inhibitors was reported [3]. The national registries suggest a small but significantly increased incidence of serious infection ranging from 1.2 to 2.78 times that of controls treated with methotrexate [3]. Mycobacteria, *Staphylococcus aureus*, *Listeria monocytogenes*, varicella zoster virus, and *Leishmania* species repeatedly appear in the case report literature and should be in the mind of the clinician faced with a serious infection in a patient with an unknown pathogen who is being treated with etanercept, infliximab, or adalimumab [5]. *Staphylococcus aureus* represented the most frequent causative pathogen and was mostly associated with bones and/or joints infections and with a worse outcome compared to that observed with other bacterial pathogens. Bacterial infections seem to occur early, within the first 6 months after the initiation of TNF-α inhibitor therapies [12, 13]. There is no evidence for an increased rate of staphylococcal carriage among anti- TNF-α treated patients [14].

TNF-α inhibitors are applied in underlying diseases such as rheumatoid arthritis, psoriasis, Crohn’s disease, and polyarteritis nodosa. The 38 % of patients had received etanercept, 34 % were received with infliximab, whereas the repartition of patients treated with TNF-α inhibitors was 51 % for etanercept, 31 % for infliximab in the whole study population [1]. The most frequent pathogen was *Staphylococcus aureus*. The pathogen was, in our case, *Staphylococcus aureus*, which showed the frequent infection secondary to receiving TNF-α inhibitors. The most common sites of secondary infection were bones and joints. Other secondary sites of infections were urinary tract, lungs, digestive tract, dental roots, muscles, and the central nervous system [1]. The cardiac involvement found in our case is very rare.

## Conclusions

This case shows the risk of severe bacterial endocarditis from the initiation of TNF-α inhibitor therapy even in a small dose, probably due to drug-induced immunological insufficiency. Physicians should be aware of secondary infections in the application of TNF-α inhibitors, even with proper usage.
